# News and Views (3&4): AAPPS-DCMP Report; 2022 CPS Prizes; JPS Award and Prize; New presidents of AIP and KPS

**DOI:** 10.1007/s43673-023-00078-3

**Published:** 2023-04-17

**Authors:** AAPPS Bulletin

**Affiliations:** Association of Asia Pacific Physical Societies, Pohang, South Korea

## AC^2^MP 2022 Meeting/Conference Report by AAPPS DCMP

The Asia–Pacific Conference on Condensed Matter Physics (AC^2^MP) 2022 took place in Sendai, Japan in a hybrid format on November 21–23, 2022. The second annual meeting was jointly organized by the AAPPS-Division of Condensed Matter Physics (DCMP) and the Institute for Materials Research, Tohoku University. In 2022, the 15th Asia Pacific Physics Conference (APPC15) was held in the summer. Consequently, AC^2^MP focused on three main topics: applications of extreme conditions for condensed matter physics, quantum beam applications in condensed matter physics, and topology-related phenomena in matter.

The total number of registered participants was 148 and 52 participants attended the meeting in person. 56% of the onsite attendants were from Japan, 19% from the Republic of China, and 8% from Indonesia.


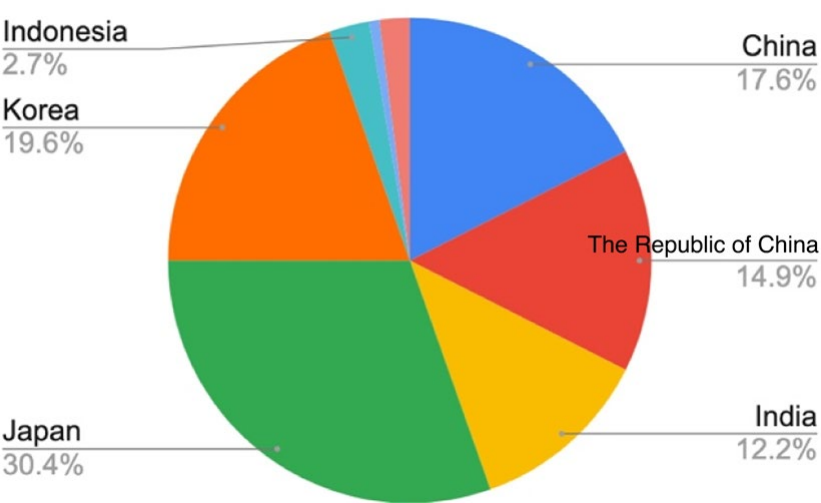


[Statistics of registered participants]

The conference started on the morning of November 21, 2022, with an opening address by the DCMP chair, Prof. Park, followed by a welcoming speech from Tohoku University and a greeting from Prof. Tajima, the president of the Physical Society of Japan. It was very memorable to have two presidents of the respective physical societies of Japan and the Republic of China attend, as the societies are responsible for the current and future AC2MP meetings.

### Day 1: Program and General Assembly

There were three sessions: Advances in Magnetic and Dielectric Systems, Optical Response and Exotic Magnetic States, and Novel Functionality in Matter. On the first day, 7 invited and 11 contributed speakers covered a broad range of current topics in condensed matter physics with several key concepts in common, including quantum phase transitions, topology, frustration, non-collinear structures, non-linear responses, and half-metals.

In the evening, the General Assembly of the DCMP was convened. The activity report and the election results for the next chair and vice chairs were approved. Prof. Kao and Prof. Ma presented the proposal for AC^2^MP 2023 to be held in Hualien, the Republic of China, from November 27–29, 2023. The certificate was conferred by the DCMP chair to Prof. Kao.


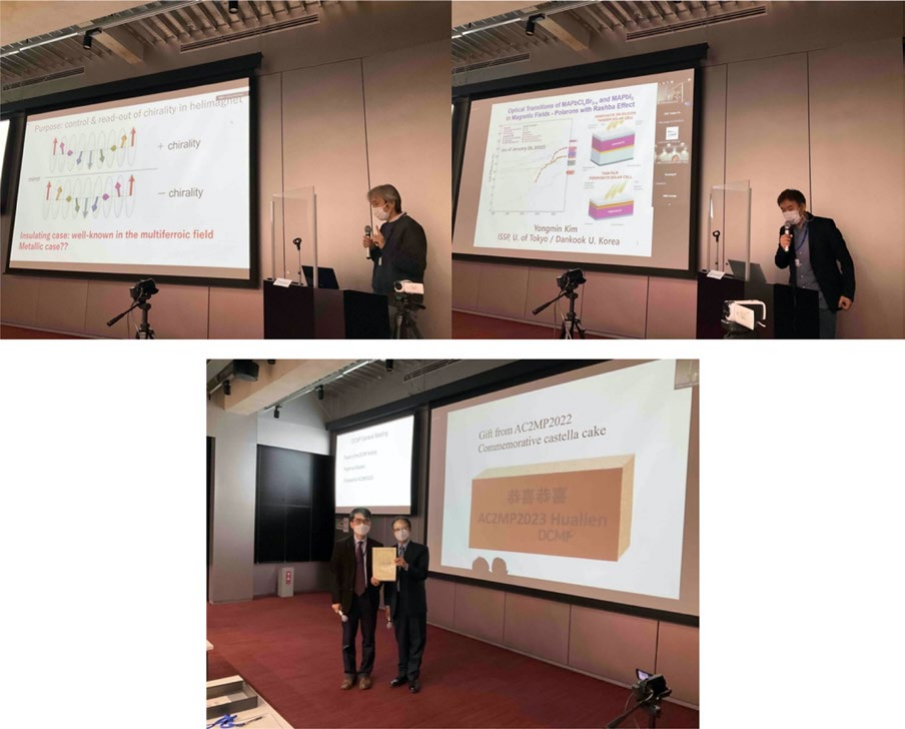


### Day 2: Program and Poster Session

November 22, 2022, there were two sessions about superconductivity, charge density wave (CDW) and nanoprobes, and the physics of thin films and layered materials. Then, a poster session with short verbal presentations was held. In the superconductivity session, high-*T*_c_ superconductivity and novel superconductive materials were discussed. In the film and layered materials session, up-to-date reports on topological matters and semiconductors were given. The poster presentations from young researchers and students showed the power and ingenuity of the next generation of physicists.


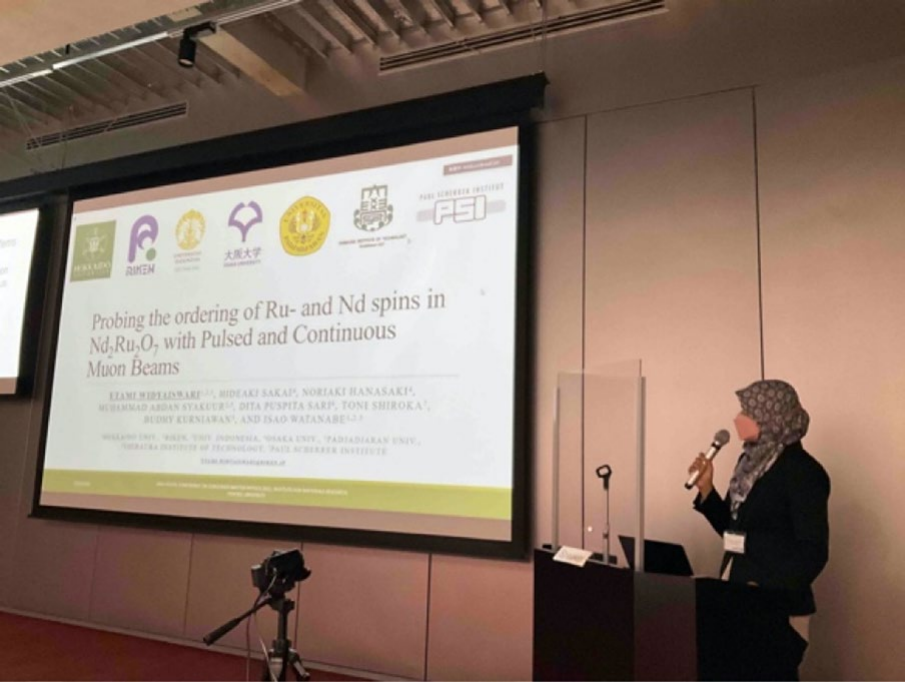


### Day 3: Program and Poster session

On November 23, 2022, three sessions on topological matters and surfaces, nano science and novel transport, quantum matters, and extreme conditions were held. The discussion centered on topology and transport as well as novel states induced by strong magnetic fields. The relation between topological matter and strong correlations was also one of the highlights of the sessions.

### Closing

In the closing remarks, the conference chair Prof. Nojiri expressed deep gratitude to attendees, program committee members, the local organizing committee, and sponsors for their support and contributions. This was the first DCMP held onsite in the post-Covid-19 era, and it was quite successful.


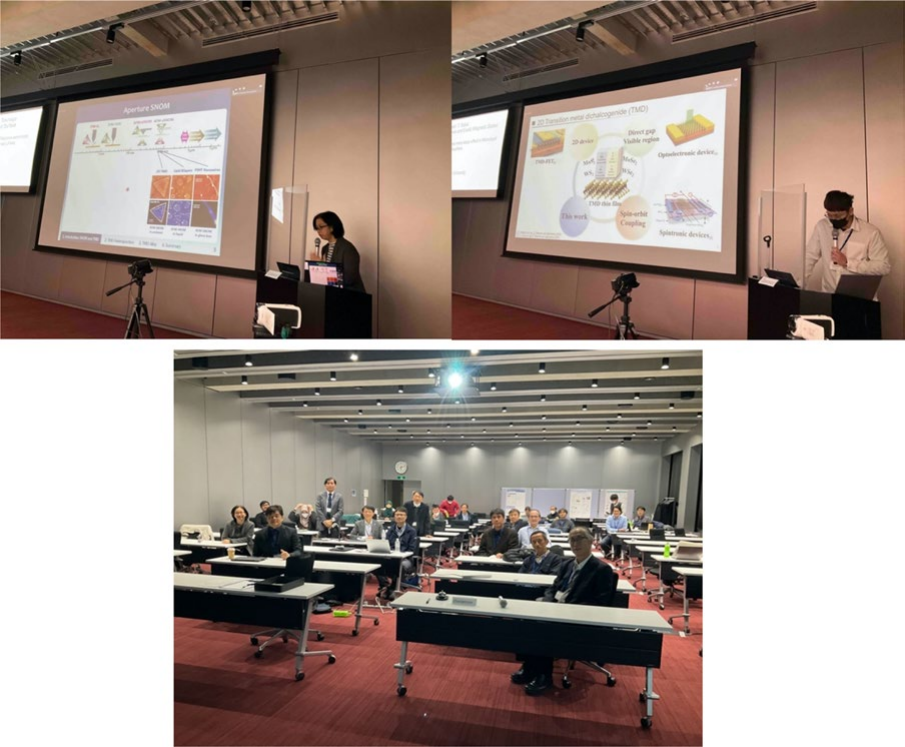


## 2022 Prizes Awarded by Chinese Physical Society by Hong-Hao Zhang (CPS)

At the 2022 fall meeting of the Chinese Physical Society (CPS) held in Southern University of Science and Technology, 5 prizes were awarded to 9 physicists. They are

- 1. The 2022 HU Gang-fu (HU Kang-fuh) Prize (for Experimental Techniques) Award by Chen Hui (The Associate Researcher at Institute of Physics CAS). 
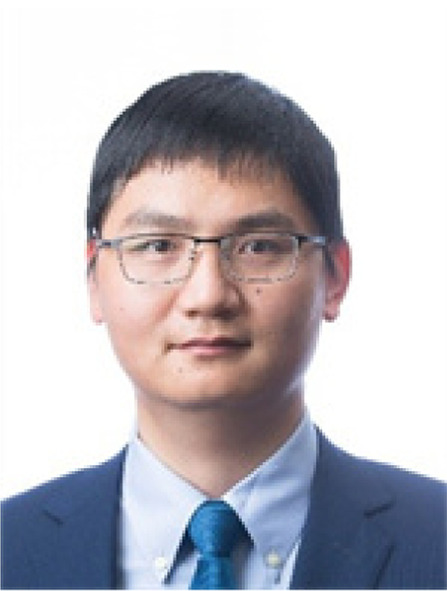
- 2. The 2022 HU Gang-fu (HU Kang-fuh) Prize (for Experimental Techniques) Award by Yang Jian-cheng (Member of the Institute of Modern Physics, Chinese Academy of Sciences). 
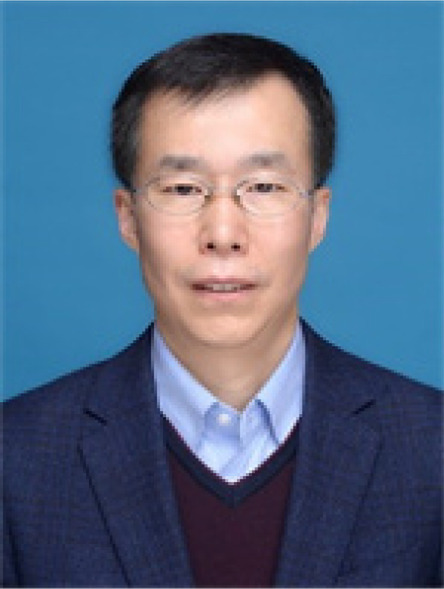
- 3. RAO Yu-tai (YAO Yu-tai) Prize (for Optics, Acoustics, Atomic and Molecular Physics) Award by Wu Jian (Professor of the State Key Lab of Precision Spectroscopy, East China Normal University) 
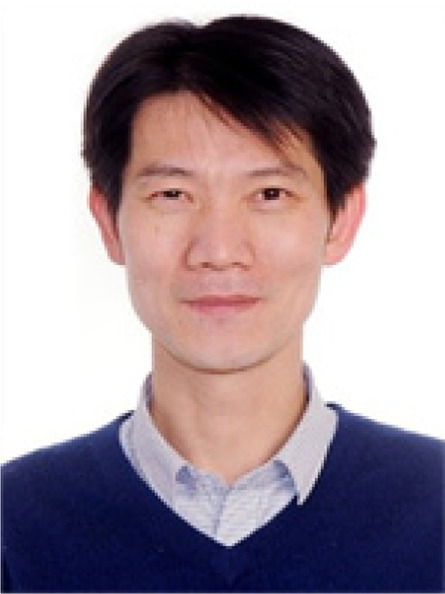
- 4. RAO Yu-tai (YAO Yu-tai) Prize (for Optics, Acoustics, Atomic and Molecular Physics) Award by Xu Xiu-Lai (Professor of Peking University, School of Physics) 
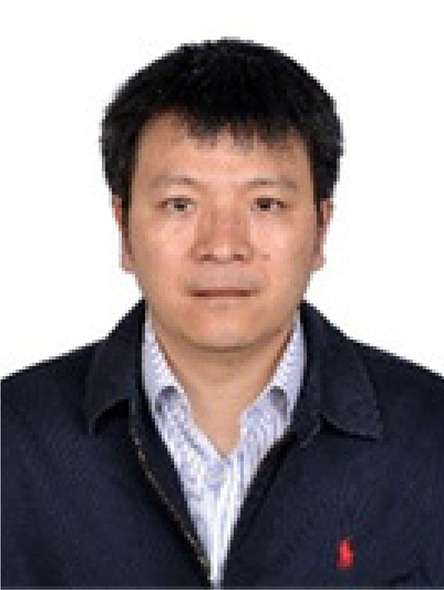
- 5. YE Qi-sun (YEH Chi-sun) Prize (for Condensed Matter Physics) Award by Yuan Hui-Qiu (Professor of Zhejiang University, School of Physics) 
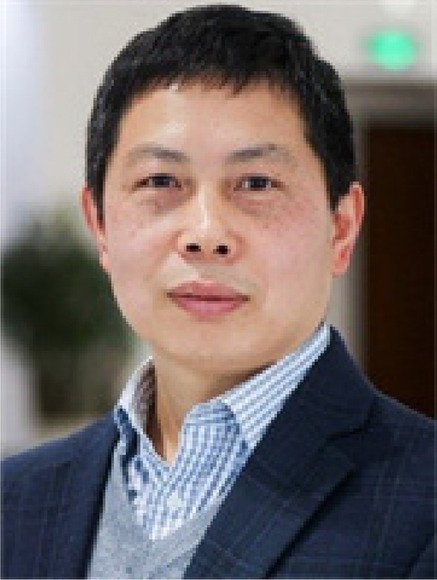
- 6. YE Qi-sun (YEH Chi-sun) Prize (for Condensed Matter Physics) Award by Yao Hong (Professor of Tsinghua University, Institute for Advanced Study) 
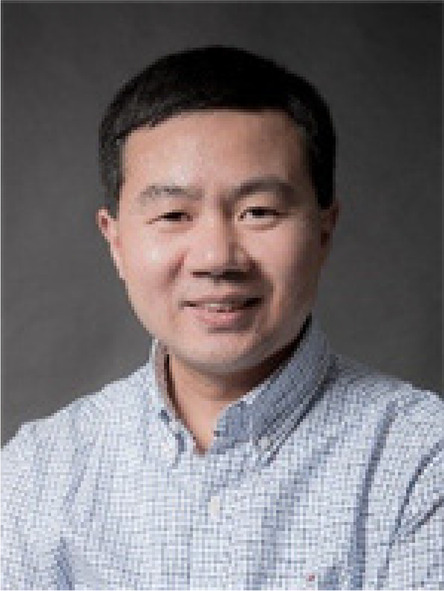
- 7. WU You-xun (WOO Yui-hsun) Prize (for Nuclear Physics) Award by Wei Long (Researcher of Institute of High Energy Physics, Chinese Academy of Sciences) 
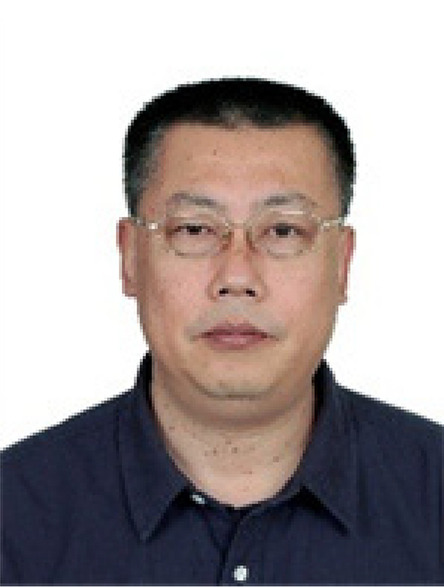
- 8. WU You-xun (WOO Yui-hsun) Prize (for Nuclear Physics) Award by Liu Yu-Xin (Professor of Institute of Theoretical Physics, School of Physics, Peking University) 
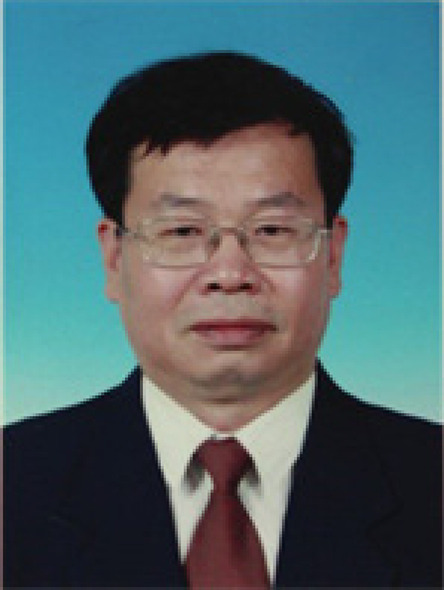
- 9. WANG Gan-chang (WANG Kan-chang) Prize (for Particle Physics and Inertial Confinement Nuclear Fusion) Award by Li Hai-bo (Researcher of Institute of High Energy Physics, Chinese Academy of Sciences) 
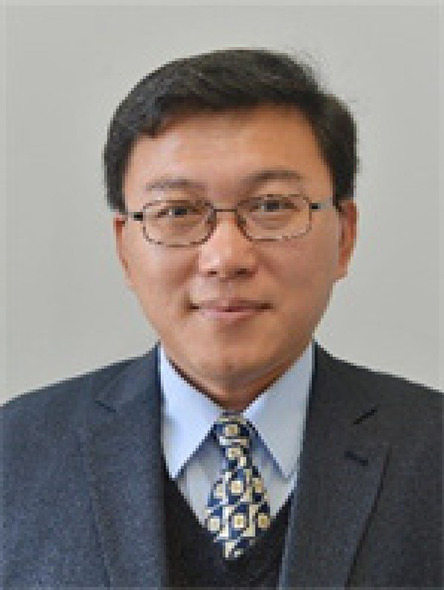

The detailed citations can be found at http://www.cps-net.org.cn/Reward/Content/show/id/4313.do

## The Physical Society of Japan Announces the Recipients of the 28th Outstanding Paper Award by JPS

In recognition of important achievements toward progress in physics, the Physical Society of Japan (JPS) annually selects outstanding papers from among original research articles published in the *Journal of the Physical Society of Japan*, *Progress of Theoretical Physics*, *Progress of Theoretical and Experimental Physics*, and *JPS Conference Proceedings*. The selection committee has chosen four papers for the 2023 award based on 18 nominations (for 17 papers) made by the editors of the JPS journals and representatives of the 10 divisions of JPS.

The award ceremony for the 28th Outstanding Paper Award will be held at noon on September 18, 2023.

The titles of the four selected papers, together with their citations, follow below.

Magnetic Properties of Layered Itinerant Electron Ferromagnet Fe_3_GeTe_2_

J. Phys. Soc. Jpn. 82, 124,711 (2013)

Bin Chen, JinHu Yang, HangDong Wang, Masaki Imai, Hiroto Ohta, Chishiro Michioka, Kazuyoshi Yoshimura, and MingHu Fang

This paper is a pioneering report on the experimental growth of a single crystal of Fe_3_GeTe_2_, which has recently been the subject of intense research as a two-dimensional itinerant ferromagnet, and on its various physical properties. The authors determined the basic properties of Fe_3_GeTe_2,_ such as its magnetic phase transition and magnetic fluctuation. The critical behavior of the magnetic properties of Fe_3_GeTe_2_ are due to strong spin fluctuations near the ferromagnetic critical point, reflecting the fact that it is a layered compound weakly coupled by van der Waals interactions, and is well explained by Moriya’s self-consistent renormalization (SCR) theory and Takahashi’s theory developed for quasi-two-dimensional ferromagnets. Furthermore, from the analysis of the Rhodes-Wohlfarth ratio, which is a measure of itinerant and localized properties, it is concluded that Fe_3_GeTe_2_ is a quasi-two-dimensional itinerant magnet.

Recently, Fe_3_GeTe_2_ has attracted much attention as one of the few materials that exhibit itinerant ferromagnetism in monolayer systems. Monolayer materials have been intensively studied by the international research community in recent years since the discovery of graphene, and van der Waals layered compounds are one of the main material groups in this field. This paper led to the discovery of Fe_3_GeTe_2_ as a monolayer itinerant ferromagnet, and its contribution to the research of various physical properties is also appreciated. The pioneering work, focusing on two-dimensional itinerant ferromagnets and its significant influence on recent studies, is now recognized for its high scientific importance. Therefore, this paper deserves the Outstanding Paper Award from the Physical Society of Japan.

Gapless Spin-Liquid Phase in an Extended Spin 1/2 Triangular Heisenberg Model

J. Phys. Soc. Jpn. 80, 093,707 (2014)

Ryui Kaneko, Satoshi Morita, and Masatoshi Imada

Quantum spin liquids, i.e., the phase of a quantum spin system in which the spin is not frozen at absolute zero temperature, have attracted much attention since Anderson proposed the idea in the 1970s. In the early stages of research, the Heisenberg model on the frustrated triangular lattice was considered to be a candidate, but later studies revealed that the ground state of the triangular lattice Heisenberg model with only the nearest-neighbor interaction is antiferromagnetic.

Although the search for quantum spin liquids has continued since then, their existence has not been established due to difficulties in numerical approaches, except for examples with exact solutions such as the Kitaev spin model. In this paper, the authors report the quantum spin liquid phase in the J1-J2 model on a triangular lattice with next-nearest-neighbor interaction in addition to the nearest-neighbor interaction when the ratio of J2 to J1 is in the region between 0.1 and 0.135, using a many-variable variational Monte Carlo method for up to relatively large sizes.

The results presented in this paper have had a strong impact on the study of quantum spin liquids and triggered many related studies. The theoretical model discussed in this paper is still recognized as one of the most promising candidates for quantum spin liquids. It is of great academic significance that this paper suggests a nontrivial quantum spin liquid phase not in a special model like the Kitaev spin model but in a relatively simple spin model. Therefore, this paper deserves the Outstanding Paper Award from the Physical Society of Japan.

Multiple Superconducting Phases and Unusual Enhancement of the Upper Critical Field in UTe_2_

J. Phys. Soc. Jpn. 89, 053705 (2020)

Dai Aoki, Fuminori Honda, Georg Knebel, Daniel Braithwaite, Ai Nakamura, DeXin Li, Yoshiya Homma, Yusei Shimizu, Yoshiki J. Sato, Jean-Pascal Brison, and Jacques Flouquet

This paper reports the multiple superconducting phases of the recently discovered superconductor, UTe_2_. The authors performed precise measurements of magnetoresistance, specific-heat, and magnetocaloric effect under high pressures. Their high-quality data made it possible to demonstrate that UTe_2_ possesses multiple superconducting phases. Such phases are akin to the superfluid phases of ^3^He. In addition, they found that some of the phases exhibit upper critical fields that exceed the Pauli limit. These results strongly suggest that UTe_2_ has spin triplet superconductivity with complex superconducting order parameters. This is an outstanding achievement that deserves the Outstanding Paper Award of the Physical Society of Japan.

Can an off-axis gamma-ray burst jet in GW170817 explain all the electromagnetic counterparts?

Prog. Theor. Exp. Phys. 2018, 043E02

Kunihito Ioka and Takashi Nakamura

The gravitational wave event GW170817, associated with the merger of two neutron stars observed on August 17, 2017, was a historic event that heralded the beginning of multi-messenger astrophysics by observing not only gravitational waves but also electromagnetic counterparts across all wavelength ranges from gamma rays to radio waves. In particular, the observed gamma rays supported a 30-year-old hypothesis that binary neutron star mergers produce short-duration gamma-ray bursts (GRBs). However, the luminosity was three or four orders of magnitude lower than standard one. In this paper, which was released on the same day as the LIGO-Virgo press conference, the authors proposed an off-axis model that naturally explains why the luminosity was lower; this model is now widely accepted as the standard model. This model gives a simple but consistent explanation of the observational results, in which the collimated emission from the relativistic jet appears many orders of magnitude fainter when observed from a direction different from the jet axis. Although different models were proposed shortly after its discovery, the authors’ off-axis model was eventually proven correct when apparent superluminal motion was discovered in long-duration observations of radio waves. The authors have been proposing the possibility of GRBs being observed from off-axis since the early 2000s, and their many years of original research came to fruition in this event. In the future era of multi-messenger astrophysics, the off-axis model of GRBs will be used as the standard model to interpret observational results and will continue to play an important role. This paper is therefore worthy of the JPS Outstanding Paper Award.

## The Physical Society of Japan: 4th (2023) Fumiko Yonezawa Memorial Prize by JPS


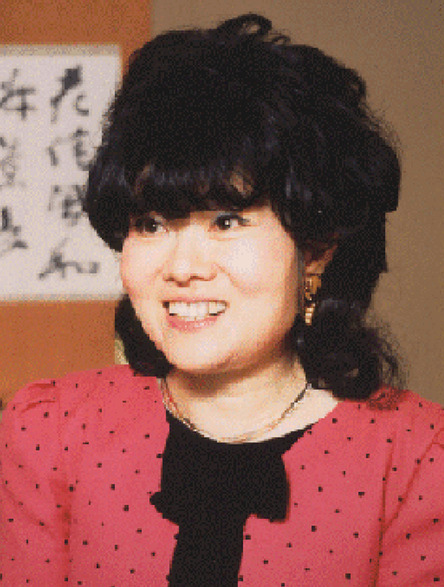


Fumiko Yonezawa

1938–2019

The late Fumiko Yonezawa, emeritus professor of Keio University, made major contributions to physics, such as the development of the coherent potential approximation, and the theory of the metal–insulator transition in liquid selenium. Prof. Yonezawa served as the first female president of the Physical Society of Japan (JPS) and as the president of the Society for Women Scientists for a Bright Future, she also promoted women scientists.

JPS has established the “Fumiko Yonezawa Memorial Prize” to celebrate the achievements of Prof. Yonezawa and to honor and encourage the activities of the women who are members of JPS.

A few prize winners will be selected once a year, with a maximum of about five recipients. The prize ceremony will be held during the annual meeting of JPS. The prize recipients will give commemorative lectures at JPS meetings within a period of one 1 year after receiving the prize. Winners will receive items such as certificates and honorary shields, as well as additional prizes, namely: (1) paid attendance fees for JPS meetings for the next 3 years; and (2) an exemption, of up to 200,000 JPY (yen), from publication fees and open access fees for the Journal of the Physical Society of Japan, and from the article processing charges for the journal, Progress of Theoretical and Experimental Physics (valid for 3 years for submissions after the prize is received).

The citations of the winners of the 4th (2023) Fumiko Yonezawa Memorial Prize are listed below. 
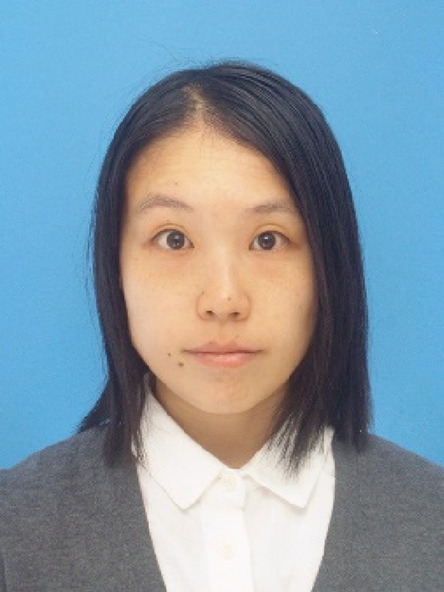


Rina Takagi

Assistant Professor, Institute of Engineering Innovation/Department of Applied Physics, The University of Tokyo

**Exploration of new materials for multi-orbital correlated electron systems showing unique magnetic states**

Dr. Rina Takagi has conducted a series of studies about strongly correlated electronic materials having multiple orbitals, revealing rich physics due to the critical role of multiple orbitals by clarifying the relation between their characteristic magnetic structures and multiple orbitals. First, she studied organic solids of single-component molecular conductors *M*(tmdt)_2_. Depending on the degeneracy between the orbitals of metallic ions *M* and the orbitals of organic ligand tmdt, she has found that various kinds of strongly correlated electronic phases appear such as paramagnetic metal, antiferromagnetic or non-magnetic Mott insulators, and orbital-selective Mott insulators. Second, she studied the second group of multiple-orbital strongly correlated materials, such as itinerant magnets of hexagonal Y_3_Co_8_Sn_4_ and cubic EuAl_4_ crystals. She has found multiple-Q ordered magnetic structures due to magnetic frustration. She especially has found that even under space-inversion symmetry, multiple-Q ordered magnetic structures such as triangular lattices of magnetic vortices are formed due to four-body magnetic interaction mediated by conduction electrons. These achievements by Dr. Takagi, revealing rich physics of multiple-orbital strongly correlated systems, are benefitting the Fumiko Yonezawa Memorial Prize of the Physical Society of Japan.


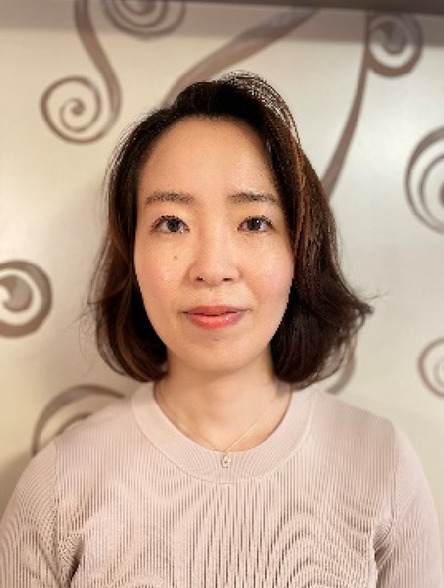


Nayuta Takemori

Specially Appointed Associate Professor, Center for Quantum Information and Quantum Biology, Osaka University

**Exploring strongly correlated many-body effects in unique electronic systems: a theoretical study**

Focusing on an interplay between peculiar electronic structures and strong correlation effects that may occur in real materials, Dr. Takemori has theoretically studied emergent phenomena with special emphasis on mass imbalance systems, quasicrystal systems, and iron-based superconductors. In particular, she is highly regarded as a leading young researcher in the theoretical studies of strongly correlated quasicrystals.

Dr. Takemori first investigated a mixed system of cold Fermi atoms with different masses. She focused on a unique degree of freedom called mass imbalance, which characterizes the mixed cold atom system, and obtained the results strongly suggesting the possibility of an emergent supersolid state in which density waves and superfluid coexist. The theoretical study, conducted thereafter, on the strong correlation effects in quasicrystal systems is representative of Dr. Takemori's research work, which was inspired by the quantum criticality in the Au–Al–Yb quasicrystal discovered in 2012. Using the dynamical mean field theory, she systematically analyzed characteristic phenomena originating from strong correlation effects and the peculiar geometrical structure inherent in the quasiperiodic systems, and thus elucidated that the phase transitions such as the Mott transition and the superconducting transition are uniquely determined even in the quasiperiodic systems, while the distribution of the local electronic quantities reflects the quasiperiodic structure below the transition temperature. Furthermore, the result obtained by extending this analysis to superconductivity turns out to be consistent with the superconducting state discovered later for the Al–Mg–Zn quasicrystal, and it gives a signature to distinguish superconductivity in periodic systems and that in quasicrystals. More recently, she has vigorously conducted research on novel physical properties arising from the unique Fermi-surface shape in iron-based superconductors and has obtained the results that provide an important step for clarifying the common properties of iron-based superconductors.

These observations lead us to conclude that Dr. Takemori’s scientific achievements deserve the Fumiko Yonezawa Memorial Prize of the Physical Society of Japan.


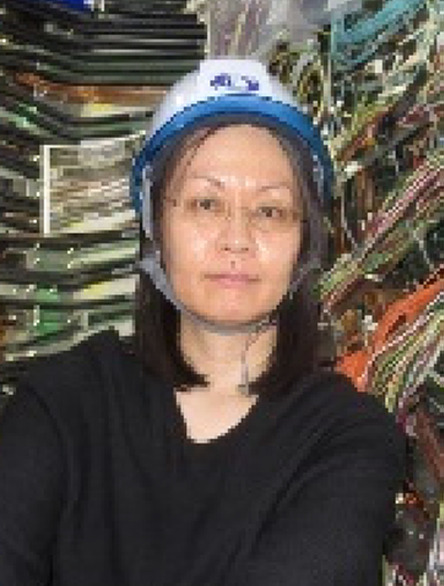


Nanae Taniguchi

Associate Professor, Institute of Particle and Nuclear Studies (IPNS), High Energy Accelerator Research Organization (KEK)

**Exploration of new physics beyond the Standard Model of elementary particles**

The purpose of the KEK Belle II experiment is to study new physics beyond the Standard Model in particle physics by producing many B0 B0-bar meson pairs by electron–positron collisions. Because the collision rate in Belle II is several ten times higher than the previous Belle experiment, many detectors had to be rebuilt.

Nanae Taniguch received her PhD for the study of new particle physics through rare B-meson decays. After receiving the degree, she started working on a project to build a new Central Drift Chamber (CDC; 2.2 m in diameter and 2.3 m in length) to measure tracks from the collision point in a high-rate environment in Belle II. Taniguchi played a central role in the development, mass-production, and installation of new electronics for CDC. She also worked on the construction of CDC and successfully read out 14,000 channels of signals as designed. She also worked on coordinating domestic and international collaborators to develop and mass-produce the electronics, and fulfilled the responsibility of its performance evaluation and quality assurance. In addition, she expanded her coverage to the trigger and data acquisition systems, and made all the detector components work together as a unified system. For her such significant contributions to the experiment, she was chosen to be the leader of the CDC group in 2019. In addition, for her contributions on solving the problems on CDC and safe operation of CDC, she received the Belle II Best Achievement Award in 2021.

As a member of the Future Planning Committee of the Japan Association of High Energy Physicists, she is actively involved in discussing and planning the future of the particle physics field. She is also actively reaching out to the public and telling the joy of working on science through science camps for female high school students and public lectures.

Her achievements and various activities stand out in the field, and encourage other female scientists to follow her. Nanae Taniguchi thus deserves to receive the Fumiko Yonezawa Memorial Prize of the Physical Society of Japan.

## Professor Nicole Bell Named Australian Institute of Physics President by AIP


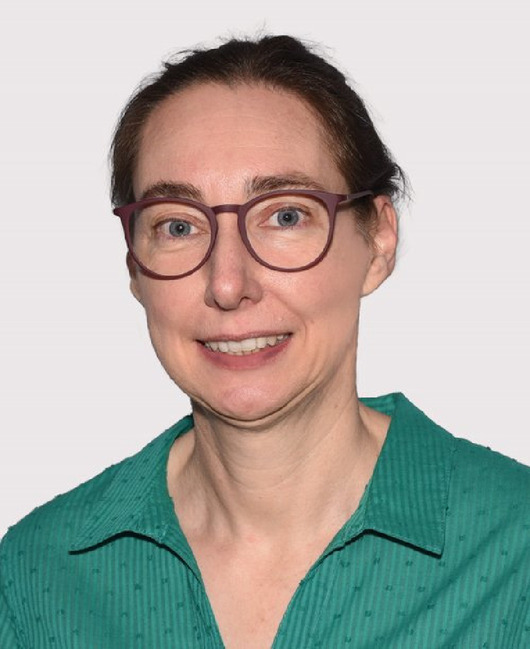


Nicole Bell, President of the Australian Institute of Physics

University of Melbourne Professor Nicole Bell has been appointed President of the Australian Institute of Physics (AIP). The AIP is the national professional body for Australian physics, dedicated to promoting the role of physics in research, education, industry and the community. This appointment recognises Professor Bell’s leadership and contributions to the discipline.

“I am honoured to represent the Australian physics community and look forward to advocating on behalf of our discipline. The AIP provides a key avenue to promote the value of science in general, and physics in particular, to government, policy makers, and the wider community.”

In her 2-year term as AIP president, she intends to promote the importance of fundamental research.

“Physics is a critical enabling science. History tells us that many technological developments were driven by fundamental research for which the eventual commercial applications were not originally anticipated. It is essential that basic breakthrough research is well supported, so that we feed the pipeline of ideas that ultimately lead to broad applications.”

Professor Bell’s own research lies at the intersection of particle physics, astrophysics and cosmology, with particular focus on dark matter and neutrino physics. She leads the Theory Program of the ARC Centre of Excellence for Dark Matter Particle Physics.

Professor Bell joined the School of Physics at the University of Melbourne in 2007. Her previous roles include postdoctoral appointments at Fermilab and Caltech in the USA. She has held an ARC Future Fellowship and was a member of the ARC Centre of Excellence for Particle Physics at the Terascale. She is a Fellow of the American Physical Society and received the Nancy Millis Medal from the Australian Academy of Science in 2020.

## Greetings from President Suklyun Hong, the Newly Elected President of the Korean Physical Society by KPS


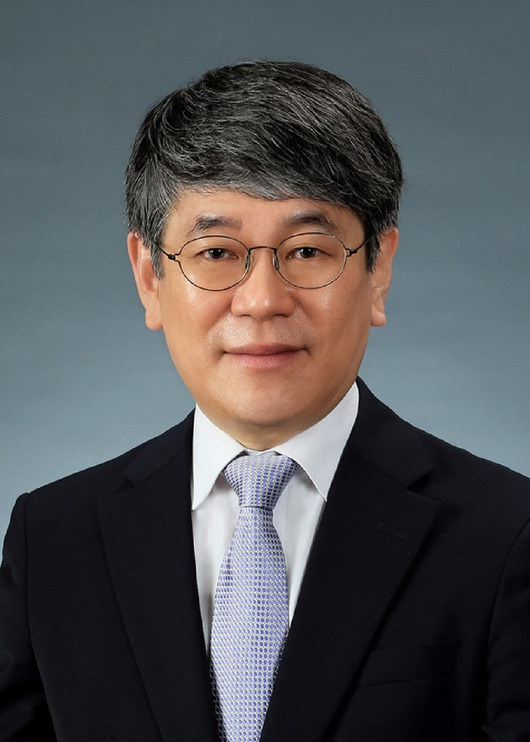


Suklyun Hong, President of Korean Physical Society

As of 2023, Professor Suklyun Hong from Sejong University has taken on the role of the 30th president of the Korean Physical Society (KPS) for a two-year term. The current executive body of the KPS includes Executive Vice-President, Professor William Jo (Ewha Womans University); Secretary of General Affairs, Professor Yunsang Lee (Soongsil University); and Treasurer, Professor Keun-Young Kim (GIST). During the New Year’s ceremony meeting held in January 2023, President Hong shared his vision and mission statement for KPS members.

Despite the challenges posed by the global COVID-19 pandemic, the Korean Physical Society (KPS) has made significant progress in recent years. However, we are aware that many hurdles still lie ahead. During the campaign period for last year's KPS presidential election, I had the opportunity to speak with many members who have shared their concerns with me. I assure you that I will do my utmost to address these issues and work towards a better future for our society. To tackle the various challenges that we face, we must unite our collective knowledge and strengths. This year is pivotal for the success of our society, and we must approach it with a sense of urgency and purpose. As the 30th president of the KPS, I am committed to promoting several exciting missions in the coming years.

Our first project aims to create a solid foundation for the renaissance of physics in Korea. As a basic science, physics education needs to be strengthened and secured as a priority. We plan to achieve this by working with the government and the national assembly to strengthen legislative policy. We will also establish easy access programs for physics education with new formats and contents that will raise awareness of physics among parents and students. To take measures to reform university education and expand education projects, we will launch the “Commission for Physics Education Initiative (CPEI)”. Through the CPEI, we will efficiently link existing educational projects and implement new ones that are suitable for the internet environment. The first project under the CPEI is the Physics League, which will be held on February 18, 2023. With this project, we plan to inherit and progressively expand the “physics certification system” and the “junior high school physics competition” to create a new physics education platform. We will continue to develop more educational projects in the future.

Second, we are committed to improving the research environment for KPS members. To achieve this goal, we will continue to expand basic research funds and distribute them in a balanced manner. Our aim is to secure sustainable research funding in the field of physics for KPS members by stabilizing the “field-specific support system”, while also expanding basic research funding. By doing so, we hope to establish a research-rich ecosystem, where young researchers can quickly establish their laboratories, and mid-career researchers can ensure stable lab operations. We will also pay close attention to ensure that researchers in traditional and challenging fields can continue their research.

Third, we are committed to ensuring that KPS journals and conferences are beneficial to our members. To achieve this goal, we are launching the “KPS Publishing” and “5-year Leap Forward Project” for our society journals, with the aim of improving the citation index of the journals through an innovative editorial process. We will improve the quality and scale of KPS spring and fall annual conferences and continuously expand them to achieve world-class standards, making those led by K-Physics leading physics conferences in Asia. Additionally, we will establish a new academic support system to promote future conferences hosted by division chapters, regional chapters, and individual members. This support system will be instrumental in facilitating conferences and disseminating new research results in the field of physics.

Through these efforts, we aim to create a KPS that is truly “of the members, by the members, and for the members”. We recognize the diversity of our membership, including women physicists, researchers of government laboratories, and others, who together form the academic society within KPS. To ensure that all members have a voice, we will promote equal participation in the executive committee, enabling everyone to communicate and listen to each other’s ideas and perspectives. By creating a more inclusive and democratic society, we hope to strengthen KPS and promote its growth in the years to come.

Last but certainly not least, we humbly ask for your continued participation, interest, and warm encouragement as we strive to achieve our goals. We wish all our esteemed members and their families good health and prosperity, and we express our deep appreciation for your ongoing support. Thank you.

